# Clear cell carcinoma of the abdominal wall: A case report with a review of the literature

**DOI:** 10.1016/j.amsu.2022.104038

**Published:** 2022-06-18

**Authors:** Ahlem Bellalah, Bahaeddine Lahbecha, Olfa Zokar, Mossaab Ghannouchi, Saber Garrach, Mohamed Ben Khlifa, Karim Nacef, Moez boudokhan, Leila Njim, Abdelfatteh Zakhama

**Affiliations:** a Department of Pathology, Fattouma Bourguiba University Hospital, Monastir, Tunisia; b Department of Gynecology and Obstetrics, Monastir Maternity and Neonatology Center, Tunisia; c Department of General and Digestive Surgery, Tahar Sfar University Hospital, Mahdia, Tunisia

**Keywords:** Endometriosis, Malignant transformation, Clear cell carcinoma, Abdominal wall, Caesarean section, Case report, CCC, clear cell carcinoma

## Abstract

**Introduction and importance:**

Clear cell carcinoma of abdominal wall is a very rare and aggressive disease. It is mostly related to malignant transformation of abdominal wall endometriosis. This paper provides a new case report and a literature review of primitive abdominal wall clear cell carcinoma.

**Case presentation:**

A 45-year-old woman with a history of a two previous caesarian section presented to the outpatient department with a tumor mass evolving since 10 years in the lower right quadrant of her abdomen. Imaging studies revealed a voluminous subcutaneous mass developing at the expense of the anterior abdominal wall. Surgical resection of the mass was performed. Histopathological examination along with immunohistochemical analysis were consistent with clear cell carcinoma. Biopsies of the endometrium and ovaries were performed and were negative for malignancy. The patient underwent therefore a hysterectomy with bilateral salpingo-oophorectomy which did not reveal any disease. The diagnosis of primitive clear cell carcinoma of the abdominal wall was then confirmed.

**Clinical discussion:**

Primitive clear cell carcinoma of the abdominal wall is an extremely rare form of cancer with usually poor prognosis. Clinicians must be aware of the possibility of malignancy of any swelling mass occurring near or within a caesarean section scar.

**Conclusion:**

Reporting more such cases is still needed to further progress in the understanding of this malignancy in addition to the development of treatment strategies.

## Highlights

• Clear cell carcinoma (CCC) occurring in the anterior abdominal wall is an extremely rare but aggressive tumor.
• It is usually secondary to malignant transformation of abdominal wall scar endometriosis.
• Caesarean section is the major factor of developing abdominal wall scar endometriosis.
• Treatment is mainly based on extensive surgery with free margins.

## 1 Introduction

Clear cell carcinoma of the female genital tract (CCC) is a well-known highly aggressive type of cancer. It can arise in the ovary, endometrium, cervix and vagina, as well in peritoneal and other extrapelvic sites [1]. Nevertheless, CCC originating in the abdominal wall is extremely infrequent. In fact, after the first case reported in 1986, only about thirty cases have been documented to date [2,3]. In addition, the development of the disease is attributed to malignant transformation of scar endometriosis [4].

This case report has been reported in line with the SCARE Criteria [5].

## 2 Case report

A 45-year-old woman presented to the outpatient department with a lump in the lower right quadrant of her abdomen that had been evolving since 10 years. She also complained of exacerbating cyclic abdominal pain, which was not associated with fever, vomiting, diarrhea, or constipation. Her past medical history was unremarkable other than two cesarean deliveries by a Pfannenstiel incision in 2007 and 2011. No family history of cancer was mentioned.

**Fig. 1:**
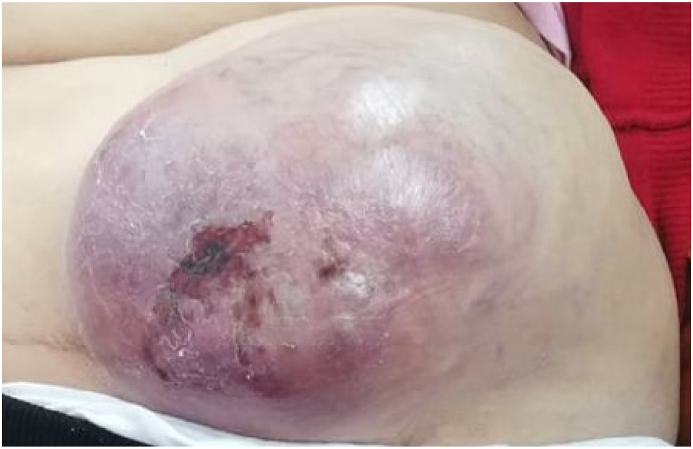
A parietal huge mass measuring 20 cm with ulcerative surface occurring in the cesarean delivery scar (arrow).

Physical examination showed a huge subcutaneous lump arising in the caesarean scar. It had a cystic consistency and showed inflammatory and necrotic cutaneous changes (Fig. 1).

**Fig. 2:**
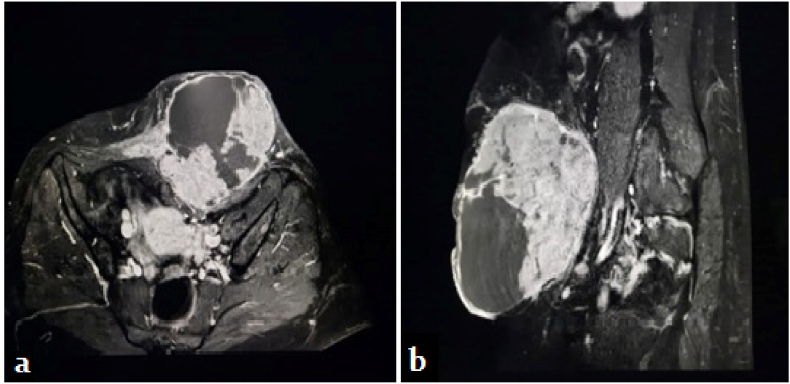
Abdominal MRI showing a subcutaneous mass developing at the expense of the anterior abdominal wall. **(a)** axial view. **(b)** sagittal view.

An abdominal MRI revealed a voluminous subcutaneous mass developing in the anterior abdominal wall and contracting a contact with the rectus abdominis muscle (Fig. 2). A biopsy was done but was inconclusive.

**Fig. 3:**
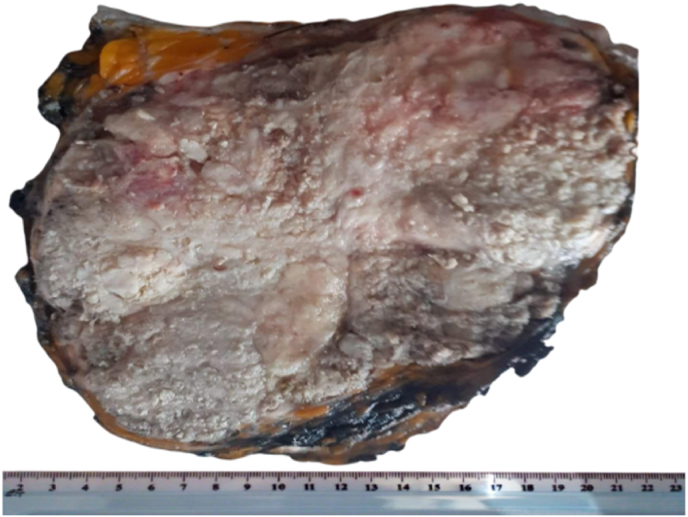
The tumor has a whitish cut section and contains foci of necrosis.

A monoblock excision of this abdominal mass was performed by an assistant professor in digestive surgery. The postoperative course was uneventful. The specimen was then sent to our pathology department for histolopathological evaluation. Macroscopic examination showed a well limited tumor of 20 × 14 × 11 cm which had a whitish cut section and contains foci of necrosis (Fig. 3).

**Fig. 4:**
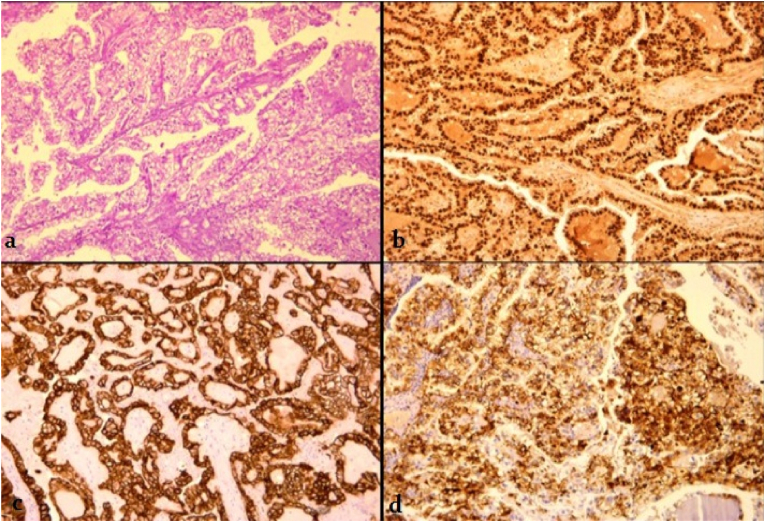
(a)The tumor is composed by papillary and tubulocystic structures lined by clear cells (H&E stain, x100). (b) Strong and diffuse immunoreactivity with PAX8 (x 100). (c) CK7 (x100) and (d) Napsin A (x100).

Histologically, the tumor consisted on a carcinomatous proliferation formed by tubulocystic and papillary structures and solid nests. Tumor cells had a clear cytoplasm and showed moderate nuclear atypia without mitosis. Foci of necrosis were extensive (Fig. 4a). Immunostaining was positive and strong with CK7, PAX8, Napsin A, patchy with PR and P53 and negative with CK20,WT-1, and calretinin (Fig. 4b-c-d). No foci of endometriosis were found. Considering histopathological and immunohistochemical findings, the hypothesis of clear cell carcinoma of the female genital tract was raised. The patient was subsequently referred to the gynecology and obstetrics department. She underwent coelioscopic and hysteroscopic exploration of the gynecologic tract which was performed by an associate professor in gynecology and obstetrics. Biopsies of the endometrium and ovaries were negative for malignancy. Afterwards, the patient underwent a hysterectomy with bilateral salpingo-oophorectomy which did not reveal any disease. The diagnosis of primitive clear cell carcinoma of the abdominal wall was then confirmed. The admission course was uneventful.

Clinical and radiological monitoring in the outpatient unit revealed the absence of recurrence and metastasis after a 12-month follow up.

## 3 Discussion

Clear cell carcinoma (CCC) occurring in the anterior abdominal wall is an extremely rare but aggressive tumor. It usually arises in abdominal scar endometriosis. The major factor of developing abdominal wall endometriosis is a previous uterine surgery, mainly caesarean section. In this context, scar endometriosis may be explained by an iatrogenic transplantation of endometrial cells to the incision during the surgical procedure [6].

Malignant degeneration of scar endometriosis is very rare with an estimated incidence of 1% [7,8]. In 1925, Sampson outlined 3 criteria to diagnose the malignant transformation of endometriosis: First, it should be a clear evidence of endometriosis foci near the tumor. Second, the histological appearance of the tumor should be compatible with an endometrial origin. Finally, other primary carcinoma sites must be excluded [9]. Later in 1953, Scott postulated a 4th criterion which emphasis the morphological demonstration of a transition between endometriosis and malignant epithelium [10].

Most frequently, endometriosis associated to malignancies takes place in the ovary. Malignant transformation has been also found in extra-gonadal sites such as intestine, rectovaginal septum, pleura and abdominal wall [11]. CCC is reported to be the most frequent histological subtype followed by endometrioid carcinoma. In a systemic review of 27 cases of cancer arising from abdominal wall, 18 patients were reported to have clear cell carcinomas [12].

Exact mechanisms of malignant transformation of endometriosis remains unelucidated. Recent studies have reported that a number of genetic alterations like loss of heterozygosity, PTEN, ARID1A, p53 and KRAS mutations have been found in both endometriosis and endometriosis-associated malignancies [13]. Emerging data links microRNAs dysregulation with ovarian carcinogenesis [14]. Other mechanisms implicate oxidative stress, hyperestrogenic environment and disturbances in immune respone [[15], [16], [17]].

In our tissue sampling, we didn't find endometrial tissue near the tumor. Despite Sampson's criteria for the diagnosis of malignant transformation of endometriosis include the coexistence of neoplastic tissue and endometriosis, some reported cases of abdominal wall clear cell carcinoma show no evidence of endometriosis foci [7,12]. This may be explained by either a sampling issue or a consequence of the total replacement of endometriosis tissue by cancer tissue.

The major complaint of abdominal wall clear cell carcinoma is a swelling abdominal mass within or near the surgical scar. Other symptoms include cyclical or permanent abdominal pain [12]. In a review of 6 cases, the mean time from the most recent pelvic surgery to diagnosis of clear cell carcinoma of abdominal wall was 20.2 years (18). There is no specific marker for malignant transformation and radiological findings are not helpful in distinguishing abdominal wall endometriosis from its degeneration [11,19]. As a result, lesions usually reach large size at the time of diagnosis with an average diameter of 11 cm [3].

Grossly, the tumor is usually presented as an ill-defined large mass infiltrating abdominal muscles. The cut surface is whitish and may show foci of necrosis and hemorrhage with cystic cavities [7**,** 20, 21]. Microscopic examination shows malignant epithelial proliferation with tubulo-cystic, papillary and solid pattern of growth. Tumors cells have abundant clear or occasionally eosinophilic cytoplasm. The clear cells lining the papillae and cystic spaces often display hobnail configuration. Papillae may show stromal hyalinization [[20], [21], [22]]. On immunohistochemical analysis, tumors cells show positivity for CK7, PAX8, Napsin A, hepatocyte nuclear factor (HNF1β) and exhibit negative staining for calretinin,WT-1, CK20,CD10, estrogen and progesterone receptors [7,22,23]. Wild-type p53 and focal p16 expression are also reported [[20], [21], [22]]. Differential diagnostics include metastasis of clear cell tumors of the female reproductive organs (ovarian, endometrial, cervical and vaginal) as well as metastasis of renal cell carcinoma, hepatocellular carcinoma and adrenal cortical tumors [23]. Other differential diagnostics include mesothelioma and urachal carcinoma [21]. An immunohistochemical panel consisting of cytokeratin AE1/AE3, CD10,CK7,CK20,and WT-1 along with clinical and radiographic information are necessary in making the distinction [23].

Giving the fact of the rarity of this type of malignancy, there is no consensus or evidence based guidelines for standardized therapeutic strategy. So that, adoption of ovarian cancer management has been usually performed. Patients are primarily treated with wide tumor resection to obtain healthy margins combined with total hysterectomy and bilateral salpingo-oophorectomy [7,11,22]. Bilateral pelvic and inguinal lymph node dissection along with infracolic omentectomy may also be performed [18] in addition to adjuvant chemotherapy and radiotherapy [3,11,18,19,22].

The prognosis of abdominal wall clear cell carcinoma seems to be poor. The median survival is about 30 months and approximately 26.5% of patients died within one year of being diagnosed [3,12].

## 4 Conclusion

CCC of the abdominal wall is very rare but usually aggressive form of cancer. It's important for clinicians to be aware of the potential malignant nature of any abdominal swelling mass occurring within or near a caesarean section scar.

## Sources of funding

None.

## Ethical approval

Exemption from ethical approval.

## Author contribution

Ahlem Bellalah and Bahaeddine Lahbecha: data analysis and writing the paper.

Olfa Zokar and Mossaab Ghannouchi contributed to Data collection, bibliography and drafting.

Saber Garrach, Mohamed Ben Khalifa, and Karim Nacef contributed to Data collection and bibliography.

Moez Boudokhan, Leila Njim and Abdelfatteh Zakhama contributed to the revision and finalization of the manuscript.

## Registration of research studies

2. Unique Identifying number or registration ID:

3. Hyperlink to your specific registration (must be publicly accessible and will be checked):

Not applicable to our case report.

## Guarantor

Dr Lahbecha Bahaeddine.

## Trial registry number

Not applicable to our case report.

## Consent

Written informed consent was obtained from the patient for publication of this case report and accompanying images. A copy of the written consent is available for review by the Editor-in-Chief of this journal on request.

## Provenance and peer review

Not commissioned, externally peer-reviewed.

## Declaration of competing interest

None.
